# Synaptic Plasticity and Learning Behaviors Mimicked in Single Inorganic Synapses of Pt/HfO_x_/ZnO_x_/TiN Memristive System

**DOI:** 10.1186/s11671-017-1847-9

**Published:** 2017-01-23

**Authors:** Lai-Guo Wang, Wei Zhang, Yan Chen, Yan-Qiang Cao, Ai-Dong Li, Di Wu

**Affiliations:** 1 National Laboratory of Solid State Microstructures and Department of Materials Science and Engineering, College of Engineering and Applied Sciences, Collaborative Innovation Center of Advanced Microstructures, Nanjing University, Nanjing, 210093 People’s Republic of China; 2 Anhui Key Laboratory of Functional Coordination Compounds, School of Chemistry and Chemical Engineering, Anqing Normal University, Anhui, 246011 People’s Republic of China

**Keywords:** Atomic layer deposition, Memristor, Pt/HfO_x_/ZnO_x_/TiN, Synapse plasticity

## Abstract

In this work, a kind of new memristor with the simple structure of Pt/HfO_x_/ZnO_x_/TiN was fabricated completely via combination of thermal-atomic layer deposition (TALD) and plasma-enhanced ALD (PEALD). The synaptic plasticity and learning behaviors of Pt/HfO_x_/ZnO_x_/TiN memristive system have been investigated deeply. Multilevel resistance states are obtained by varying the programming voltage amplitudes during the pulse cycling. The device conductance can be continuously increased or decreased from cycle to cycle with better endurance characteristics up to about 3 × 10^3^ cycles. Several essential synaptic functions are simultaneously achieved in such a single double-layer of HfO_x_/ZnO_x_ device, including nonlinear transmission properties, such as long-term plasticity (LTP), short-term plasticity (STP), and spike-timing-dependent plasticity. The transformation from STP to LTP induced by repetitive pulse stimulation is confirmed in Pt/HfO_x_/ZnO_x_/TiN memristive device. Above all, simple structure of Pt/HfO_x_/ZnO_x_/TiN by ALD technique is a kind of promising memristor device for applications in artificial neural network.

## Background

The concept of the memristor was first proposed by Prof. Chua in 1971 according to the completeness of the circuit theory [1]. It represents the relationship between magnetic flux and charge, and is considered the fourth fundamental passive circuit element beside the resistance, capacitance and inductance [1, 2]. However, it was ever just a theoretical conception until Strukov et al. found the missing memristor device in studying TiO_2_ cross array in 2008 [2], which triggers the interest of researchers around the world. Synapse is the smallest unit of learning and memory of the human brain [3], and the bionic simulation of synaptic learning is considered as an important route to realize artificial neural network. Lots of work on synapse simulation have been reported in the past; however, most research focused on ordinary electron devices using a number of transistors and capacitors to realize artificial synapse. This led to high-energy dissipation at high density and the limitation of software program running. The new memristor system is now known as the closest to the synaptic device because of its nonlinear transfer characteristics similar to the neural synapse [2].

Recently, several groups have been successfully designed and fabricated memristor devices using TiO_x_ [4], Ag_2_S [5, 6], Cu_2_S [7], Ag/Si [8], RbAg_4_I_5_ [9], InGaZnO [10], WO_x_ [11, 12], PEDOT:PSS [13], and other materials [14–19], and the spike-timing-dependent plasticity (STDP) and nonlinear transmission characteristics of the synapse have been simulated using these memristor devices. Nevertheless, because these memristor models do not involve all the synapse learning function, it is very difficult to mimic the synapse learning function accurately at present. Moreover, it is also a bottleneck to lack the high quality memristor materials and the manufacturing processing of mass memristor devices compatible with microelectronic technology, restricting the rapid development of memristor systems.

Atomic layer deposition (ALD) is a kind of novel thin film deposition technique based on unique sequential self-limited surface chemisorptions reactions [20, 21]. Since 2001 the international technology roadmap for semiconductors (ITRS) regarded ALD as candidate technology preferred for semiconductor industry along with metalorganic chemical vapor deposition (MOCVD) and plasma-enhanced CVD [22], ALD has become one of the most promising and competitive deposition approaches for microelectronics, optoelectronics, and nanotechnology due to its unique advantages such as simple and precise thickness control, excellent three-dimensional (3D) conformality, large-area uniformity, good reproducibility, and low growth temperature, especially compatibility with traditional semiconductor processing. Plasma-enhanced ALD (PEALD) using plasma species as reactants allows for more freedom in processing conditions (substrate temperature and choice of precursors) and for a wider range of materials (metal and nitride) compared with the conventional thermally driven ALD method.

Recently, an ultra-low-energy oxide-based synapse with three-dimensional vertical structure of Pt/AlO_x_/HfO_x_/TiN has been developed for implementation of robust high-accuracy neuromorphic computation systems [23]. The maximum energy consumption of less than 1 pJ per spike has been confirmed. Among them, the key resistive switching layer of AlO_x_/HfO_x_ synapse was prepared by ALD technology. In this letter, we reported a kind of new memristor with the simple structure of Pt/HfO_x_/ZnO_x_/TiN, which was fabricated completely via combination of thermal-atomic layer deposition (TALD) and PEALD. The synaptic plasticity and learning behaviors of Pt/HfO_x_/ZnO_x_/TiN memristive system have been investigated deeply.

## Methods

**Fig. 1:**
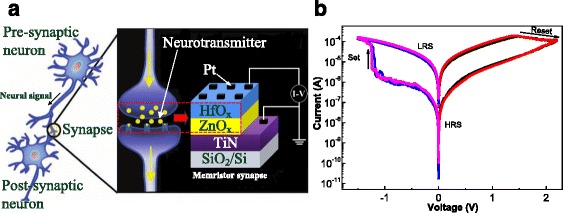
Schematic of the memristor device of Pt/HfO_x_/ZnO_x_/TiN and its *I*–*V* characteristics. **a** Analogy between the biological synapse and the electronic synapse based on the Pt/HfO_x_/ZnO_x_/TiN memristor device. **b** *I*–*V* characteristics of the Pt/HfO_x_/ZnO_x_/TiN synapse device measured by a typical DC double sweep

A single synaptic device unit based on Pt/HfO_x_/ZnO_x_/TiN memristor was fabricated on TiN-coated Si substrates by means of combination of TALD and PEALD, as schematically shown in the Fig. 1a. P-type Si (100) wafers with a resistivity of 1~10 Ω cm were used as the starting substrates. Then, 30~60-nm-thick TiN was deposited on Si as bottom electrode at 400 °C using TiCl_4_ and NH_3_ plasma gas as the Ti and N sources by PEALD. Subsequently, 5-nm-thick ZnO and 5-nm-thick HfO_2_ thin film was deposited on TiN/SiO_2_/Si substrates at 250 °C with 30 and 50 cycles by TALD, respectively. The Zn, Hf, and O precursor were diethylzinc (DEZ), Hf[N(C_2_H_5_)CH_3_]_4_ (TEMAH), and H_2_O, respectively. Finally, 120-nm-thick Pt top electrodes were DC sputtered through a shadow mask with a diameter of 150 μm. Post-annealing was performed at 500 °C for 20 s in N_2_ using rapid thermal annealing. The electrical properties were measured under different modes using Keithley 4200-SCS semiconductor parameter analyzer, 33600A waveform generator, and an oscilloscope (TDS 2012B Tektronix) on probe station (CasCade Summit 12000 B-M). The bottom electrode was grounded, and the signals were applied to the top electrode in the measurements. The single inorganic synaptic device was detected to emulate a series of synaptic behaviors such as LTP, STP, and STDP.

## Results and Discussion

Figure 1b shows the *I*–*V* characteristics of the memristor device of Pt/HfO_x_/ZnO_x_/TiN measured by the typical DC double sweep. The initial electroforming voltage of the device is −2 V (not shown here). The sweeping voltage was applied from 0 to −1.5 V for set and 0 to 2.2 V for reset with a reading voltage of 0.1 V at room temperature. This device shows a typical bipolar resistive switching characteristic. The transition between high and low resistance states can be realized by applying the set or reset voltage. It indicates that the device conductivity has also an increasing or decreasing changes correspondingly, with the set or reset process. This phenomenon is very similar to the potentiation or depression of the signal in the biological nerve synapse [24].

The resistive switching mechanism of the device of Pt/HfO_x_/ZnO_x_/TiN is similar to a memristor model based on the electronic barrier at the Pt/TiO_2_ interface due to the oxygen vacancy drift under applied electric field proposed by Strukov’s group [2, 25]. The bilayer oxide of HfO_x_/ZnO_x_ on TiN bottom electrode is equal to the structure of TiO_2_/TiO_2-y_ on Ti/Pt one. The TiN electrode with high oxygen affinity causes a lot of oxygen vacancies in the intrinsic n-type ZnO_x_ film [26], forming oxygen-deficient layer, whereas HfO_x_ film near Pt top electrode contains richer oxygen with less oxygen vacancies. The device conductivity is dependent on the concentration distribution of oxygen vacancies at the interface of metal/oxide and the inferior to create or destroy conducting channels. The migration of oxygen vacancies between the anoxic layer of ZnO_x_ and the oxygen-rich layer of HfO_x_ under various bias electric fields changes the electronic barrier height, so the overall conductivity of the device can be adjusted and controlled. Further work is needed to confirm the influence of oxygen vacancy distribution of bilayer oxide films on resistive switching behavior.

**Fig. 2:**
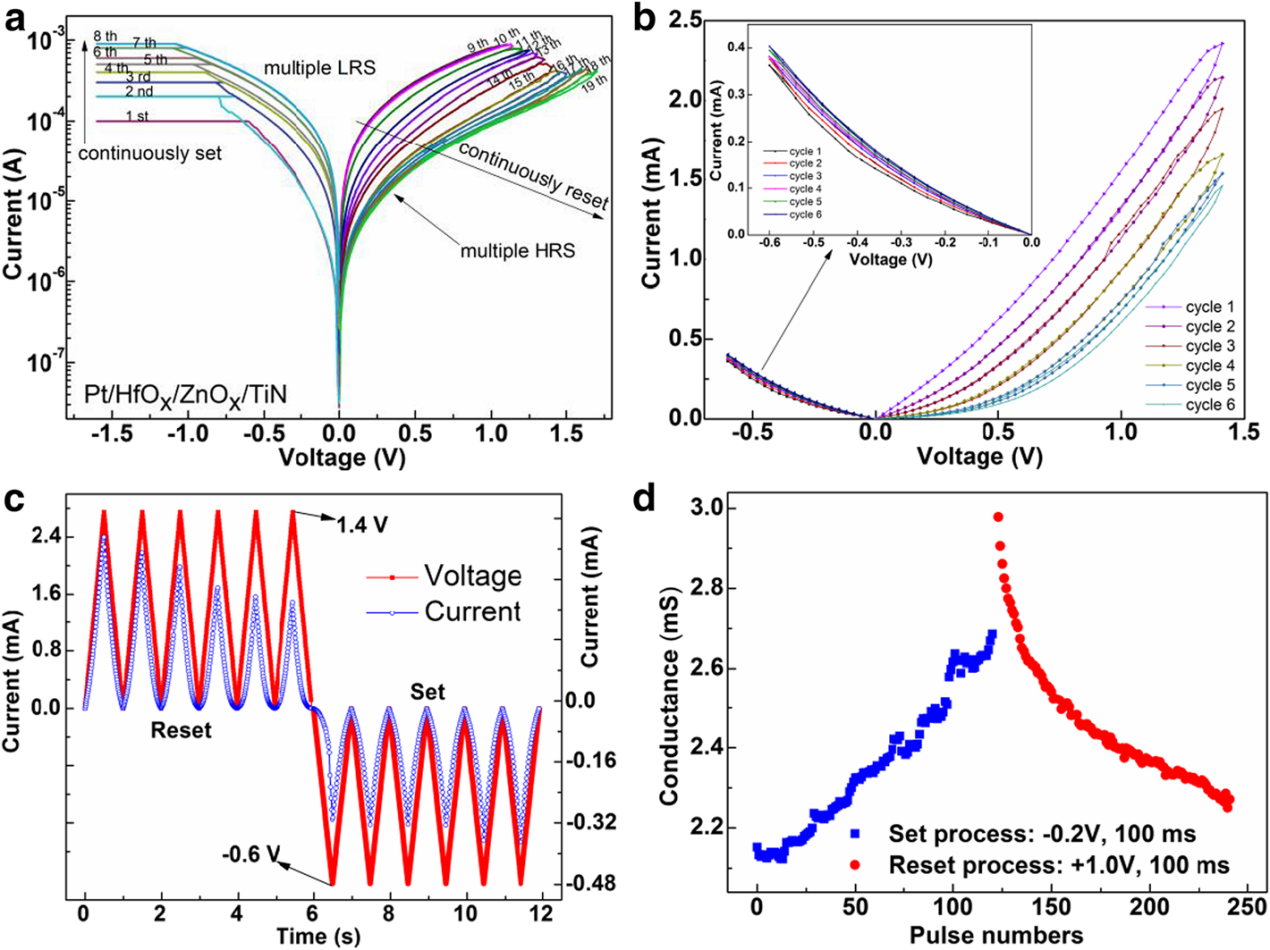
*I*–*V* characteristics of the Pt/HfO_x_/ZnO_x_/TiN synapse device and conductance dependence on consecutive depressing or potentiating pulses. **a** *I*–*V* characteristics of the Pt/HfO_x_/ZnO_x_/TiN synapse device measured by a modified DC double sweep. **b** *I*–*V* characteristics of the memristor at positive and negative bias voltages. The voltage sweep range is from 0 to 1.4 (–0.6) V then back to 0 V, and the time for a sweep cycle is 1 s. The device conductivity continuously decreases or increases during the positive or negative voltage sweeps. **c** The curves of voltage and current versus time, which are plotted from the data in (**b**). **d** The curves of device conductivity versus pulse numbers. The device conductivity can be decreased or increased by consecutive depressing or potentiating pulses

In order to emulate the functions of a nerve synapse, one multiple-state analog memory in the transition process of high and low resistance states should be obtained at first. Figure 2a shows the *I*–*V* characteristics of the device measured by a modified DC double sweep. The sweep sequence is denoted by the number in Fig. 2a. First, we performed a continuous set process by continuously increasing the compliance from 0.1 to 1.0 mA at an interval of 0.1 mA, which is equivalent to a successive enhance of the conductance. Then, a continuous reset process was carried out with a consecutive decrease of the conductance by gradually adjusting the reset voltage from 1.0 to 1.7 V at an interval of 0.05 V, which is similar to the depression of the biological synapse. Eight low resistance states (LRS) and 11 distinguishable high resistance states (HRS) have been obtained for Pt/HfO_x_/ZnO_x_/TiN synapse device during continuous set and reset process, respectively. It is worth noting that the resistance can be continuously decreased or increased between multiple intermediate states without going back to the original state. This point is crucial for electronic synapse applications [16].

The synapse device actually operates under the pulse signal rather than DC bias sweep voltage. It can be regarded as a two-terminal device with characteristics of nonlinear transmission efficiency. The connection strength between neurons determines the transfer efficiency, which can be dynamically changed with the stimulation or the suppression of the pulse signal, and maintains a continuous state change. Inspired by the memristor model [25, 26], our device consists of a double layer structure of HfO_x_/ZnO_x_ so as to realize such synapse function.

As shown in Fig. 2b, when a continuous sweep positive pulse voltage from 0 to 1.4 V is applied to the device, the conductivity decreases continuously with six easily recognized states; when a continuous sweep negative pulse voltage from 0 to −0.6 V is applied to the device, the conductivity increase continuously with difficultly distinguishable ones. In order to clearly illustrate this change trend, the curves of current and voltage versus time are plotted in Fig. 2c. Figure 2d shows the device conductivity can also be increased or decreased by consecutive potentiating or depressing pulses. It is easily observed that the conductance in the last set pulse is different from the one in the first reset pulse. This can be ascribed to the partial change in internal structure after the device has experienced a process from very low conductivity to high conductivity. As known, the migration of oxygen vacancies in oxide-based memristor leads to the conductivity change during the device operation, even if the reverse bias voltage cannot be completely restored the memristor to the initial state. This is also a common phenomenon in other memristor devices [12].

**Fig. 3:**
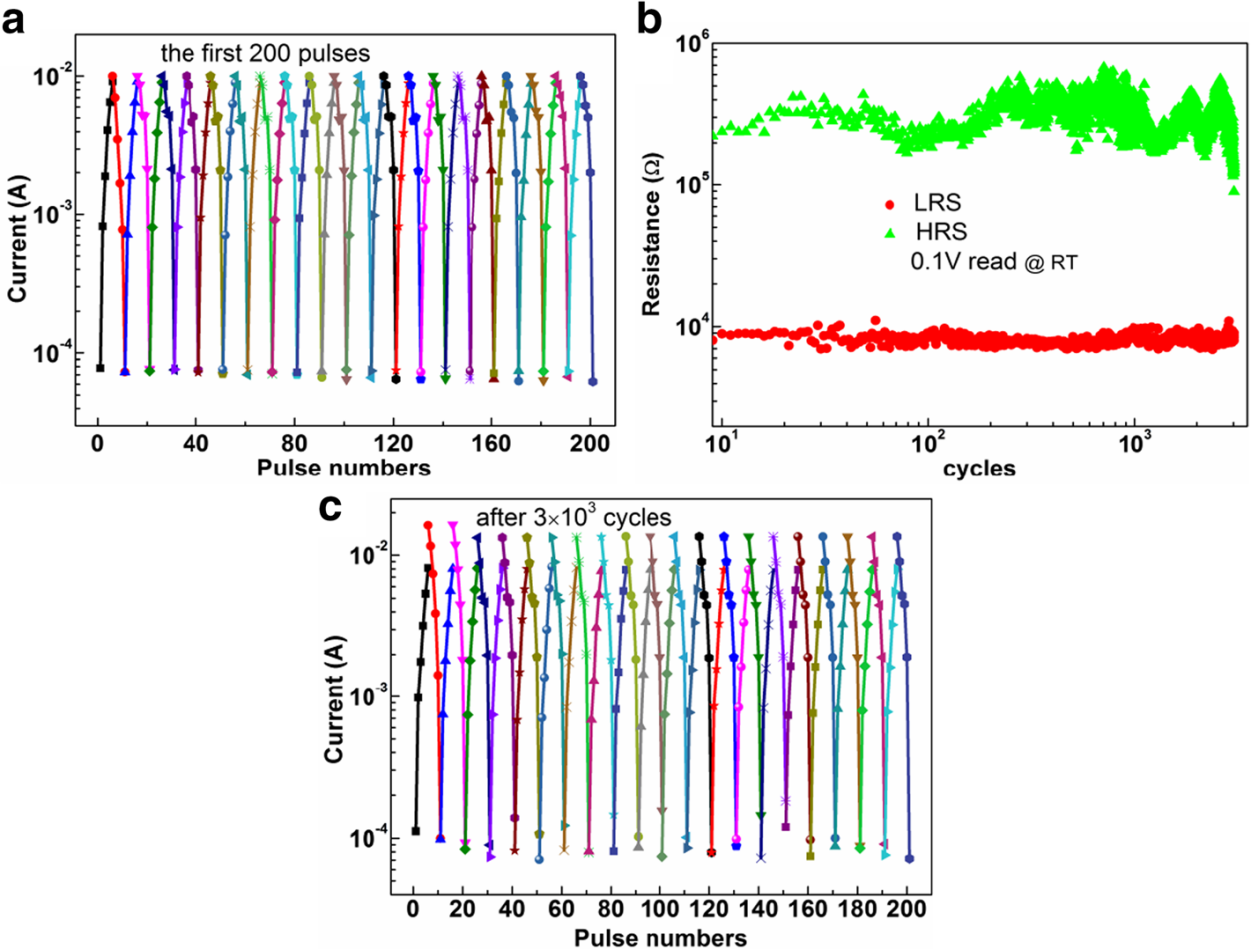
The property of gradual current change in the context of pulse cycling of Pt/HfO_x_/ZnO_x_/TiN. **a** Current evolution of the device for the first 200 pulse numbers. **b** Endurance test up to 3 × 10^3^ cycles. Pulse of *±*2 V with 100-ms width was applied to switch the device between the LRS and the HRS, and the resistances were read out at 0.1 V at room temperature. **c** Current evolution of the same device after the 3 × 10^3^ cycle endurance test in **b**

The property of gradual current change in the circumstance of pulse cycling of Pt/HfO_x_/ZnO_x_/TiN synapse device was examined. Figure 3a shows the current evolution of the first 200 pulse numbers of the device. The pulse width was fixed to be 100 ms and the gradual set process was performed from the HRS. It can be clearly seen that when the amplitude of the six pulses consecutively decreases from −0.5 to −1.0 V, the corresponding current gradually increases from the order of 10^−4^ to 10^−2^ A. Then, the gradual reset process was performed from the LRS. With the six pulses consecutively increases from 1.1 to 1.6 V, the corresponding current gradually decreases from the order of 10^−2^ to 10^−4^ A. After the dozen-pulse cycles, the device returns back to the original HRS. Similar dozen-pulse cycles were repeated for 20 times without marked change. Subsequently, the endurance test of the device using pulse voltage was carried out, as depicted in Fig. 3b. ±2 V pulse with 100 ms width was applied to switch the device between LRS and HRS with read operation at 0.1 V. The device displays a stable resistance ratio of HRS/LRS above 10 during 3 × 10^3^ cycles.

Figure 3c shows the current evolution of the same device after 3 × 10^3^ cycle endurance test of Fig. 3b. Compared to the result in Fig. 3a, the current value at the end of the last set pulse is not equal to the current one in the initial state of the first reset pulse. But, the device still retains the property of the gradual current change by consecutive potentiating or depressing pulses.

**Fig. 4:**
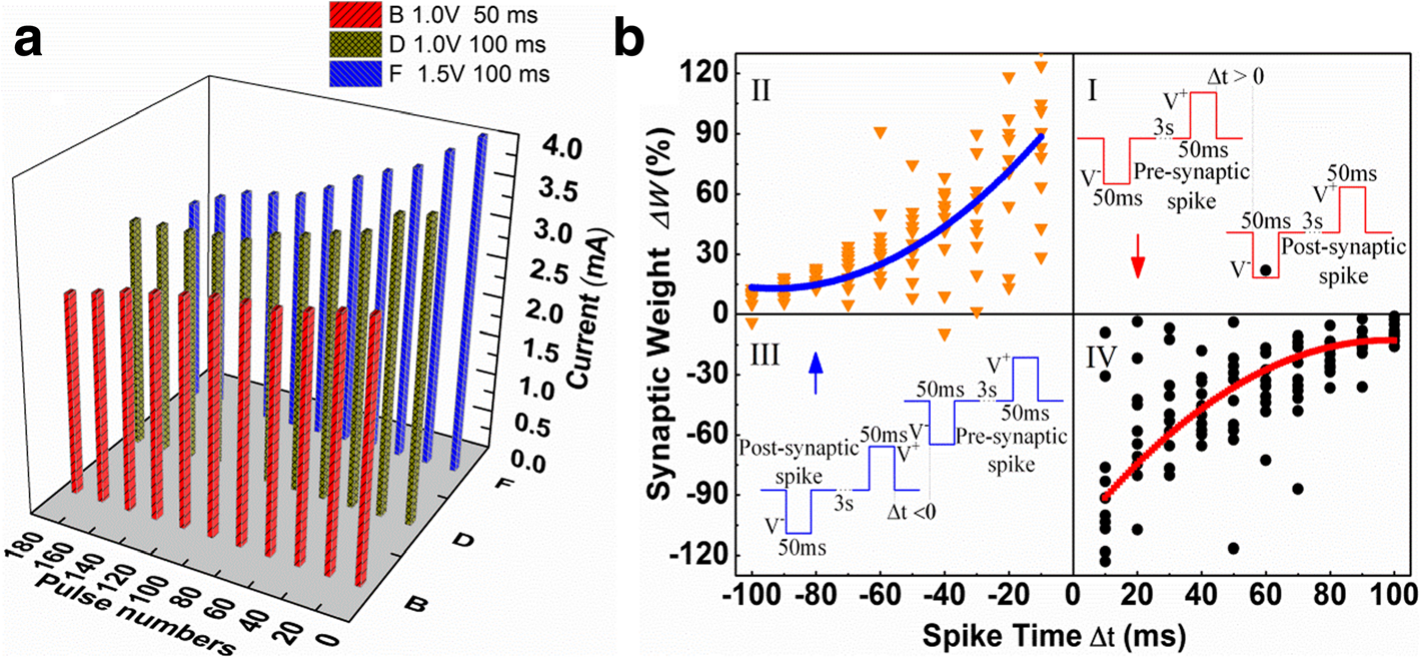
Nonlinear transmission characteristics and spike-timing-dependent plasticity (STDP) of the memristor device. **a** Response of a memristor device to different pulse programs; **b** Emulation of STDP learning rule in Pt/HfO_x_/ZnO_x_/TiN memristive device—the relative change of the memristor synaptic weight (Δ*W*) versus the relative spike timing (Δ*t*). And the solid line is the fitting exponential curve to the experimental data. The insets illustrate various spike schemes. The pulse pair comprises a positive and a negative voltage pulse with amplitude of 1.0 V and width of 50 ms. The interval between the two pulses is Δ*t* ms (*t* = ±10*n*, *n* = 1, 2, …, 10). The current compliance is not set in the whole emulation process. The current values are read at 0.1 V after 5 min of the spikes

The current increases or decreases through a continuous positive or negative pulse, as seen in Fig. 2b. Further studies have elucidated that the increasing of the pulse voltage from 1.0 to 1.5 V and the pulse duration from 50 to 100 ms can produce quite different current responses with various amplitudes and speeds, as shown in Fig. 4a. In this memristor device, if we regard the device conductivity as a synaptic weight, the above results are similar to the nonlinear transmission characteristics of the biological neural synapses. By applying positive and negative pulse voltage to stimulate and inhibit the synapse, the change of the device conductivity can be recorded as the movement of the conductive front between the double layers of thin films. When a positive voltage is applied to the top electrode of the device, the oxygen ion will migrate under the electric field and lead to the front end to form the oxygen-rich layer. The above results in Fig. 4a indicate that the device can dynamically respond to changes caused by external signals and have basic transmission properties of biological synapses.

One of the most important characteristics of the nerve synapse is its synaptic plasticity [27]. On the one hand, synaptic plasticity refers to the association between different signal stimuli in the presence of time. On the other hand, synaptic weight can be altered by pre- and post-synaptic stimulation in the spike-timing-dependent plasticity (STDP) rule. The STDP is one of important synaptic adaptation rules in the competitive Hebbian learning theory. At the same time, it is necessary to simulate the brain function in artificial neural network [28]. When the presynaptic stimulation is earlier than the postsynaptic one, the synaptic efficiency will be enhanced, resulting in long-term potentiation. On the contrary, when the postsynaptic stimulation is earlier than the presynaptic one, the efficiency is reduced, resulting in long-term depression. Meanwhile, the change of synaptic weights in STDP has a close relationship with the relative time of presynaptic/postsynaptic stimulus. It also depends on the frequency of the signal stimulus, i.e., the time interval between different stimuli. In the above two points, there exists significant similarity between the memristor device and synapse.

In the Pt/HfO_x_/ZnO_x_/TiN device, the Pt/HfO_x_ and the ZnO_x_/TiN as the presynaptic membrane and the postsynaptic membrane, respectively, as illustrated in Fig. 1a. In order to use the STDP rule, we designed a set of pulse signal, as shown the insets I and III in Fig. 4b. A pair of signals, including the amplitude of the V^−^/V^+^ = ^−^1.0 V/1.0 V pulse signal as a presynaptic and postsynaptic spikes, was applied to the top electrode and the bottom electrode, respectively. In the design of the two kinds of spike signals, the 3-s interval time between V^−^ and V^+^ is enough to ignore the influence of V^−^ on V^+^ and prevent from disturbing excitatory postsynaptic current [29]. The time interval between the final presynaptic spike and the initial postsynaptic spike is defined as the relative time of Δ*t*. When the presynaptic spike appears before postsynaptic spike, Δ*t* > 0 (Fig. 4b I); when the postsynaptic spike occurs before presynaptic spike, Δ*t* < 0 (Fig. 4b III). First the postsynaptic or presynaptic current *I*_1_ was measured as the control value, and then the spike-pair was applied to the device after 5 min. When the spike pair was over, the presynaptic or postsynaptic current *I*_2_ was measured after waiting for 5 min. According to the literature [10], the relative change of the synaptic weights (Δ*W*) is defined as (*I*_2_–*I*_1_)/I_1_. Figure 4b shows the emulation results of STDP learning rule in Pt/HfO_x_/ZnO_x_/TiN memristive device—the relative change of the memristor synaptic weight (Δ*W*) versus the relative spike timing (Δ*t*). And the solid line is the fitting exponential curve to the experimental data. It can be seen from Fig. 4b, when the presynaptic spike appears before the postsynaptic spike, synaptic weights will increase; when the presynaptic spike occurs after the postsynaptic spike, synaptic weights will decrease. And the smaller the Δ*t* between the two spikes, the greater the Δ*W*. STDP data points in Fig. 4b have a remarkable statistical scatter, which has also been observed in the biological synapses. As a result, the characteristics of memristor device are basically consistent with the STDP rule of the biological synapse.

According to the length of the memory time, synaptic plasticity can be classified as short-term plasticity (STP) and long-term plasticity (LTP), and which correspond to short-term memory and long-term memory in psychology. STP represents a transient connection of neurons and is generally held for a few minutes, while LTP represents a permanent connection of neurons and is generally held for a few hours to several years [6, 10, 12, 13]. In addition, the STP can be changed through repeated training to LTP, similar to the human brain memory.

**Fig. 5:**
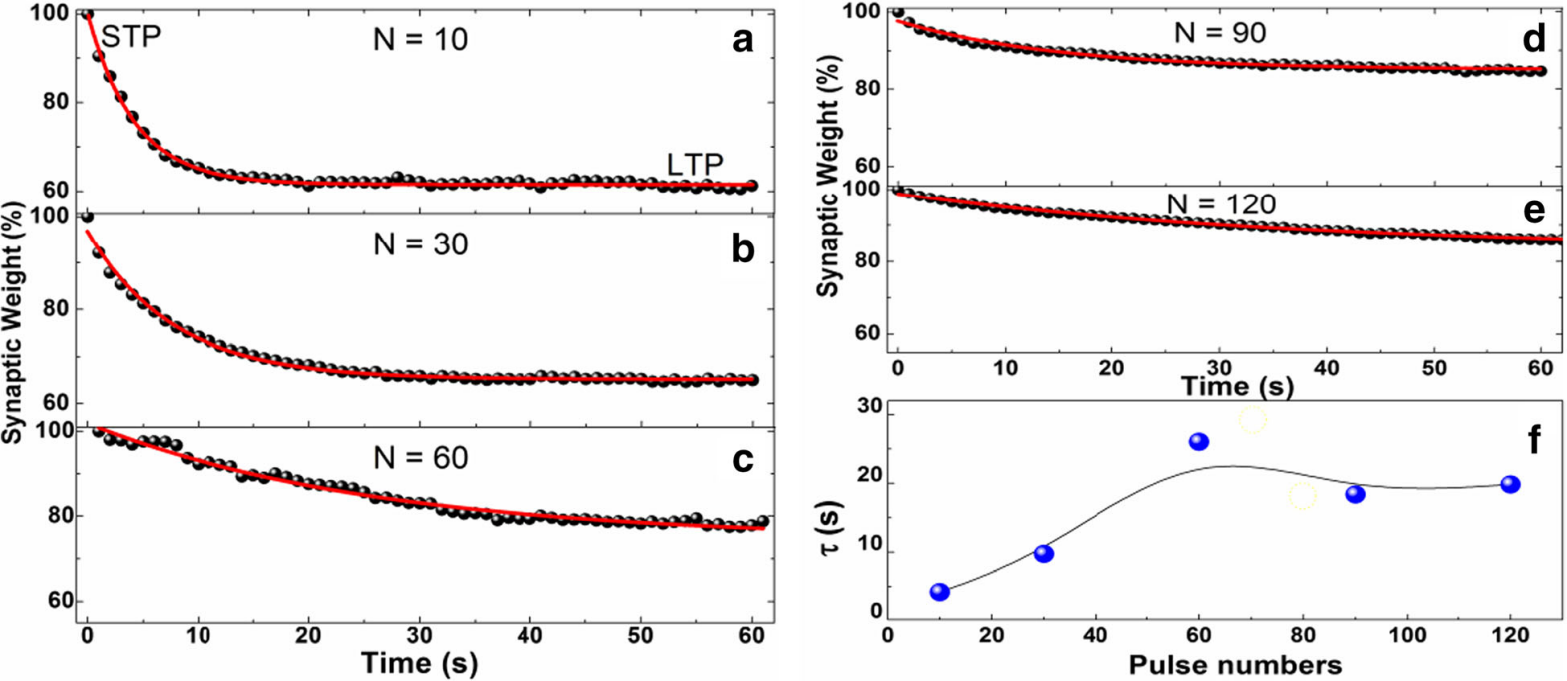
The memory retention for synaptic weight of Pt/HfO_x_/ZnO_x_/TiN memristive device: transformation from STP to LTP induced by repetitive pulse stimulation. **a**–**e** Memory forgetting process (*black solid circle* in figure) was experienced by different numbers of pulse stimulation, in which the normalized data was fitted using Eq. 1. **f** The relaxation time (*τ*) changes with the number of pulses by fitting the data from Fig. 5(**a**–**e**)

In order to observe the transformation from STP to LTP, the following experiments were designed. First, a fixed width and height pulse with fixed interval was loaded to the memristor for different number of pulses (*N*). After the last one of the pulse was applied, the current value was read immediately at an interval of 1 s by using 1.0 V, 10 ms pulse voltage. During a fixed time of 61 s, the memory retention curves of Pt/HfO_x_/ZnO_x_/TiN memristive device by loading different pulse numbers (*N* = 10, 30, 60, 90, 120) are recorded in Fig. 5a–e. The results show that the synaptic weights begin to decay after the applied pulse is removed. In the beginning, the decay rate is relatively faster, corresponding to the relaxation of the STP process. Subsequently, the decay rate becomes slow, corresponding to the relaxation of the LTP process. This kind of change tendency is consistent with the memory forgetting curve of the human brain. It is worth noticing that the synaptic weights are not attenuated to the initial state but remain in the intermediate state, which means that the memory consists of two parts: transient plasticity and permanent plasticity, i.e., STP and LTP. In order to descript the memory loss of the memristor device, we used the Eq. (1) to fit the data of memory retention curve [13].

1 $$ {I}_t={I}_0+A \exp \left(-t/\tau \right) $$

where *I*_*t*_ and *I*_0_ represent the current value at time *t* and initial stable state, respectively. *A* is a constant related to the current, and *τ* is the relaxation time constant. The red curves in Fig. 5a–e are the fitting curves. With increasing the pulse number *N* from 10 to 120, the forgetting rate of the device decreases from 40 to 15%. When the applied pulse number *N* is more than 90, the forgetting rate tends to saturation with a constant value of 15%. The above results also elucidate that the forgetting rate becomes slow with repeated stimulations, and the memory retention increases with repeated stimulations. This phenomenon indicates that the memory can be changed from STP to LTP through repeated training and learning [6, 12].

Figure 5f shows the relaxation time (*τ*) versus the pulse number from the data fitting in Fig. 5a–e. The relaxation time constant *τ* has a definite significance, which can be used to assess memory forgetting rate. Using Eq. 1 to fit the STP process, the estimated value of *τ* ≈ 17.7 s can be obtained. When *t* < 17.7 s, the synaptic weight decreases rapidly with increasing the pulse number; when *t* > 17.7 s, the synaptic weight increases slowly with increasing the pulse number.

## Conclusions

In summary, a kind of new memristor with the simple structure of Pt/HfO_x_/ZnO_x_/TiN was fabricated completely by TALD and PEALD. The synaptic plasticity and learning behaviors of Pt/HfO_x_/ZnO_x_/TiN memristive system have been investigated deeply. Multilevel resistance states are obtained by varying the programming voltage amplitudes during the pulse cycling. The device conductance can be continuously increased or decreased from cycle to cycle with better endurance property up to about 3 × 10^3^ cycles. Several essential synaptic functions are simultaneously achieved in such a single double-layer of HfO_x_/ZnO_x_ device, including nonlinear transmission characteristics such as LTP, STP, and STDP. The transformation from STP to LTP induced by repetitive pulse stimulation is confirmed in Pt/HfO_x_/ZnO_x_/TiN memristive device. Above all, simple structure of Pt/HfO_x_/ZnO_x_/TiN by ALD technique is a kind of promising memristor device for applications in artificial neural network.

## Abbreviations

ALD: Atomic layer deposition
LTP: Long-term plasticity
PEALD: Plasma-enhanced ALD
STDP: Spike-timing-dependent plasticity
STP: Short-term plasticity
TALD: Thermal-ALD
